# New achievements: from submission to disclosure

**DOI:** 10.1590/2176-9451.20.1.015-016.edt

**Published:** 2015

Dreams determine what you wish. Action determines what you achieve. Aldo Novak, Brazilian journalist.

We have recently announced a minor change in the editorial process of Dental Press Journal of Orthodontics (DPJO).^1^ All manuscripts submitted to the journal are now sent for initial analysis carried out by one of our associate editors. Should the associate editor, in conjunction with the editor-in-chief, decide that the article is of low level of priority, it will be immediately sent back to the author. Conversely, should initial analysis suggest that the article is suitable for publication, it will continue on the flow of submission and it will be thoroughly analyzed by a group of three to four consultants. This initial scrutiny not only aims at examining the article's originality and whether or not it is in accordance with the standards of the journal and institutional review boards, but also aims at assessing the quality of the English language in which it is written. 

Such *modus operandi *has been implemented thanks to our new submission system. Noteworthy, we are pleased to announce that Dental Press Journal of Orthodontics has been operating through ScholarOne Manuscripts, a platform that enables authors, reviewers and editors to work effectively. ScholarOne is a submission system used by different periodicals around the world; therefore, it is more familiar to foreign authors and reviewers. It is an important step forward for the progressive internationalization process of DPJO.^2^

Internationalization is an extension of one's world view, it means removing the flags that set backyards apart. In this context, the electronic library SciELO (Scientific Electronic Library Online) has recently announced new criteria for articles to be submitted in its platform with a view to contributing to the internationalization of Brazilian scientific journals. The following shall be analyzed: indicators related to the percentage of articles published in English and authors of foreign affiliation, in addition to the increased proportion of foreign researchers working as associate editors and referees. DPJO has been published in English since 2010. Since then, it has also increased the number of foreign reviewers. The number of foreign authors has exponentially increased as well,^2^ especially after the journal was indexed by MEDLINE.^3^ Even before we were acquainted with the new criteria set by SciELO, we already sought foreign reviewers who were able to contribute to the growth of the orthodontic sciences by means of DPJO. Presently, we are pleased to announce a new achievement: affiliating our first foreign associate editor, Prof. Carlos Flores-Mir, from the University of Alberta - Canada. It is an honor for any periodical from around the world to be able to count on the expertise of a researcher of such high level. 

Development in scientific production requires that periodicals be indexed in databases of which purpose is to make information available to clinical and scientific communities, quickly and systematically. In the mid-2013, we proudly announced that Dental Press Journal of Orthodontics became part of the select collection of MEDLINE/PubMed, the most important database providing references to health sciences information. Now, we are pleased to announce another major achievement, the next step. Late 2014, Dental Press Journal of Orthodontics received the final approval to be included in PubMed Central (PMC), a respectable database, a reservoir of scientific articles. Thus, full-text articles published in DPJO will be available online through PubMed Central. Making the content of a given journal available at PMC provides numerous advantages. One of which is noteworthy: worldwide visibility provided by the journal full access. Such accessibility was already made possible by SciELO. Now, it will be enabled by PMC and shall be rendered an incommensurable impulse. 

Applause be given to Dental Press unselfish spirit towards science for gratuitously sharing DPJO entire content. PMC is an excellent tool that can be used to set the articles published by DPJO wide open to the world, thereby extending its international visibility and the impact produced by its publications. Another achievement accomplished as a result of high-quality Brazilian Orthodontics with eyes, ears and voice on the watch of the world.

David Normando - editor-in-chief (davidnormando@hotmail.com)
