# Supplementary material for: New achievements: from submission to disclosure
Source: Dental Press J Orthod. 2015 Jan-Feb;20(1):15–6. doi: 10.1590/2176-9451.20.1.015-016.edt (PMC4373010; doi:10.1590/2176-9451.20.1.015-016.edt)
Supplement: Translation

# Translation: Novas conquistas: da submissão à divulgação

Sonhos determinam o que você quer. Ação determina o que você conquista. Aldo Novak, jornalista brasileiro.

Há pouco tempo, anunciamos uma pequena alteração no processo editorial do *Dental Press Journal of Orthodontics* (DPJO)^1^. Todos os trabalhos submetidos passam a ser encaminhados para uma análise inicial, a ser realizada por um dos nossos editores-associados. Se o editor-associado, consubstanciado pelo editor-chefe, decidir que o artigo possui um baixo nível de prioridade, esse será imediatamente devolvido ao autor. Por outro lado, se a avaliação inicial sugerir que o estudo apresenta potencial para publicação, o manuscrito seguirá seu fluxo de submissão e será detalhadamente analisado por um painel de três ou quatro consultores. Esse escaneamento inicial visa examinar a originalidade do artigo, sua adequação às normas da revista, cumprimento das normas dos comitês de ética e a qualidade do inglês. 

Esse *modus operandi* está sendo implementado graças ao nosso novo sistema de submissão. Destarte, temos o prazer de anunciar que o *Dental Press Journal of Orthodontics* está operando através do ScholarOne Manuscripts, uma plataforma que permite aos autores, revisores e editores um trabalho de maior eficiência. O ScholarOne é um sistema de submissão utilizado em periódicos do mundo inteiro; portanto, mais familiar aos autores e revisores de outros países. Um importante passo no progressivo processo de internacionalização do DPJO^2^. 

A internacionalização é uma amplificação da visão de mundo, a remoção das bandeiras que separam quintais. Nesse compasso, a biblioteca eletrônica SciELO (Scientific Electronic Library Online) anunciou, recentemente, novos critérios para indexação em sua plataforma, com o objetivo de contribuir para a internacionalização dos periódicos científicos produzidos no Brasil. Serão examinados indicadores relacionados à evolução da porcentagem de artigos publicados em inglês e de autores com afiliação estrangeira, além do aumento da proporção de pesquisadores de outros países que exerçam as funções de editores associados e pareceristas. Já desde 2010, o DPJO é publicado em inglês, assim como vem ampliando o número de revisores estrangeiros. O número de autores estrangeiros também cresce exponencialmente^2^, principalmente após a indexação pelo MEDLINE^3^. Antes mesmo do conhecimento desses novos critérios, já buscávamos revisores estrangeiros que aceitassem contribuir para o crescimento da ciência ortodôntica, por meio do DPJO. Nesse momento, temos o prazer de informar mais uma conquista, a inclusão de nosso primeiro editor-associado estrangeiro, o Prof. Carlos Flores-Mir, da Universidade de Alberta-Canadá. Uma honra, para qualquer periódico mundial, poder contar com a expertise de um pesquisador desse nível. 

O crescimento da produção científica tem exigido a indexação dos periódicos em bases de dados, com o objetivo de tornar a informação visível à comunidade clínica e científica, de forma rápida e sistemática. Em meados de 2013 anunciamos, prazerosamente, que o *Dental Press Journal of Orthodontics *passava a integrar o seleto acervo da MEDLINE/PubMed, a mais importante base de dados com informações das ciências da saúde^3^. Agora, temos a satisfação de informar mais uma importante conquista, o passo seguinte. O *Dental Press Journal of Orthodontics*, no final de 2014, recebeu a aprovação final para inclusão na PubMed Central (PMC), respeitável base de dados para depósito de artigos científicos, um repositório. Dessa forma, textos completos dos artigos publicados no DPJO estarão eletronicamente disponíveis por meio da PubMed Central. Existem diversas vantagens em disponibilizar o conteúdo de uma revista científica no PMC. Uma merece destaque: a visibilidade mundial, por meio da acessibilidade de todo o conteúdo do periódico. Essa acessibilidade já era possível por meio da SciELO e agora, por meio da PMC, ganhará um incomensurável impulso. 

Aplausos ao espírito altruísta da editora Dental Press para com a ciência, ao compartilhar, gratuitamente, todo o conteúdo do seu periódico. A PMC é uma excelente ferramenta para escancarar ao mundo os trabalhos publicados no DPJO, ampliando a sua visibilidade internacional e o impacto das suas publicações. Mais uma conquista alavancada pela qualidade da Ortodontia brasileira, com olhos, ouvidos e voz na espreita do mundo.

David Normando - editor-chefe (davidnormando@hotmail.com)
